# Uncoordinated Transcription and Compromised Muscle Function in the *Lmna*-Null Mouse Model of Emery-Dreifuss Muscular Dystrophy

**DOI:** 10.1371/journal.pone.0016651

**Published:** 2011-02-22

**Authors:** Viola F. Gnocchi, Juergen Scharner, Zhe Huang, Ken Brady, Jaclyn S. Lee, Robert B. White, Jennifer E. Morgan, Yin-Biao Sun, Juliet A. Ellis, Peter S. Zammit

**Affiliations:** 1 The Randall Division of Cell and Molecular Biophysics, King's College London, New Hunt's House, Guy's Campus, London, United Kingdom; 2 Centre for Ultrastructural Imaging, King's College London, New Hunt's House, Guy's Campus, London, United Kingdom; 3 The Dubowitz Neuromuscular Centre, Institute of Child Health, University College, London, United Kingdom; The National Institute of Diabetes and Digestive and Kidney Diseases, United States of America

## Abstract

*LMNA* encodes both lamin A and C: major components of the nuclear lamina. Mutations in *LMNA* underlie a range of tissue-specific degenerative diseases, including those that affect skeletal muscle, such as autosomal-Emery-Dreifuss muscular dystrophy (A-EDMD) and limb girdle muscular dystrophy 1B. Here, we examine the morphology and transcriptional activity of myonuclei, the structure of the myotendinous junction and the muscle contraction dynamics in the *lmna*-null mouse model of A-EDMD. We found that there were fewer myonuclei in *lmna*-null mice, of which ∼50% had morphological abnormalities. Assaying transcriptional activity by examining acetylated histone H3 and PABPN1 levels indicated that there was a lack of coordinated transcription between myonuclei lacking lamin A/C. Myonuclei with abnormal morphology and transcriptional activity were distributed along the length of the myofibre, but accumulated at the myotendinous junction. Indeed, in addition to the presence of abnormal myonuclei, the structure of the myotendinous junction was perturbed, with disorganised sarcomeres and reduced interdigitation with the tendon, together with lipid and collagen deposition. Functionally, muscle contraction became severely affected within weeks of birth, with specific force generation dropping as low as ∼65% and ∼27% of control values in the extensor digitorum longus and soleus muscles respectively. These observations illustrate the importance of lamin A/C for correct myonuclear function, which likely acts synergistically with myotendinous junction disorganisation in the development of A-EDMD, and the consequential reduction in force generation and muscle wasting.

## Introduction

Muscular dystrophies are a clinically heterogeneous group of diseases characterised by progressive muscle weakness and wasting of variable distribution and intensity [1]. They are subdivided into groups with respect to the age of onset, and in accordance with the primary muscle groups affected [1]. In many instances, a severe cardiomyopathy is also present, sometimes in the absence of the myopathy. The genes responsible for muscular dystrophies encode proteins that form a disparate group, both in function and location within the cell. For example, dystrophin is associated with the cytoskeleton and its absence causes Duchenne muscular dystrophy (DMD) [2], while emerin is located in the nuclear membrane and its deficiency underlies X-linked Emery-Dreifuss muscular dystrophy (X-EDMD) [3]. Also located in the nuclear envelope are lamin A and lamin C, mutations in which are responsible for autosomal-Emery-Dreifuss muscular dystrophy (A-EDMD) [4] and limb girdle muscular dystrophy (LGMD) 1B [5], in addition to several other degenerative diseases [6].

The A-type (lamin A/C) and B-type (lamin B1 and B2) lamins are type V intermediate filament proteins, which are major components of the nuclear lamina: proteinaceous network underling the inner nuclear membrane. Along with associated nuclear envelope proteins such as emerin, nesprin isoforms and SUN 1 and 2 (Sad1 and UNC84 domain-containing 1 and 2), lamins contribute to maintaining the structural integrity of the cell by cross-linking the nuclear envelope to the cytoskeletal network via the link of nucleoskeleton and cytoskeleton complex (LINC) [7]. All A-type lamins are encoded by *LMNA* by alternative splicing, and mutations in this gene give rise to 16 tissue-specific degenerative diseases collectively known as laminopathies [6]. Two non-mutually exclusive hypotheses have been proposed to explain this repertoire. The first underlines the importance of lamin A/C as structural proteins in maintaining nuclear architecture, since their absence results in sensitivity to mechanical stress [8]. The second focuses on the role of the nuclear lamina as a transcription platform, since nuclear lamins interact with a variety of transcription factors such as c-Fos [9], [10] and disruption to such interactions lead to anomalous down-stream gene expression [10].

The majority of laminopathies arise from dominant missense or frameshift mutations (e.g. [4], [11]), whereas mouse models to date, need to be homozygous for a *lmna* mutation to display a phenotype [12]. A patient reported to have a complete lack of *LMNA* function had a severe phenotype and died at birth [13], so the *lmna*-null also provides a useful model for A-EDMD [14]. *Lmna* ^−/−^ mice are viable, but exhibit growth retardation from 2–3 weeks of age, and stop growing after ∼4 weeks. At 4–6 weeks of age, the mice develop a rapidly progressive dilated cardiomyopathy (DCM), with death usually around 8 weeks [15]. By 3–4 weeks, an abnormal posture and gait develops, with many skeletal muscles exhibiting dystrophic features, including the presence of centrally located myonuclei, variability in myofibre diameter, signs of atrophy and hyaline or flocculent cytoplasm. Muscle involvement is not uniform however, with muscles of the head, tongue and diaphragm largely unaffected in mutant mice [14]. As observed in A-EDMD patients, myonuclei in *lmna*-null mice exhibit both structural and antigen distribution abnormalities (e.g. LAP2, lamin B, emerin). *Lmna* heterozygous mice do not show any overt signs of growth retardation or dystrophic muscle, but develop atrio-ventricular defects as early as 10 weeks of age [16].

Myonuclei are often analysed using muscle sections from patients and mouse models. However, this technique does not allow accurate enumeration of myonuclei per myofibre, analysis of their distribution along a myofibre, or determination of the proportion with abnormal morphology or function. To address these limitations, we examined complete isolated myofibres from mutant mice, allowing the ready scrutiny of all myonuclei and satellite cells (the resident stem cells of adult muscle [17]). We found fewer myonuclei present in myofibres from *lmna*-null mice, many of which had morphological abnormalities, with variability in size, shape and chromatin distribution. Assaying both epigenetic modifications and the transcriptional machinery, revealed distinct differences in the transcriptional activity between myonuclei within a myofibre. Myonuclei with abnormal morphology and decreased transcriptional activity were distributed along the length of the myofibre, but were particularly evident at the myotendinous junction. Indeed, the structure of the myotendinous junction was generally disturbed, with less inter-digitations between myofibre and tendon, disorganised sarcomeres and collagen/lipid deposition. Investigation of muscle contraction dynamics showed that function in *lmna* ^−/−^ muscles was dramatically impaired at 4–5 weeks of age, as shown by the reduction in muscle specific force. Thus there are clear structural myofibre abnormalities and deregulation of gene expression between individual myonuclei distributed throughout a myofibre, which likely contribute to the marked decline in muscle contractile ability observed.

## Materials and Methods

### Ethics statement

Mice were bred, and experimental procedures carried out, in accordance with British law under the provisions of the Animals (Scientific Procedures) Act 1986, under project license PPL0672, as approved by the King's College London Ethical Review Process committee.

### Mouse models

A heterozygous breeding colony of mice with a null allele of *lmna* [14] was established to obtain *lmna* ^−/−^, *lmna* ^−/+^ and *lmna* ^+/+^ (wild-type), from mice supplied by Carlos Lopez-Otin (University of Oviedo, Spain). Mice were genotyped by PCR on genomic DNA obtained from the tail using the Manual ArchivePure DNA Purification Kit (5Prime, Gaithersburg, MD, USA) with the following primers:

Forward: 5′ CGATGAAGAGGGAAAGTTCG 3′

Mutant-specific reverse: 5′ GCCGAATATCATGGTGGAAA 3′

Wild-type-specific reverse: 5′ CCATGGACTGGTCCTGAAGT 3′

Cycling parameters were 95°C/30 s, 60°C/30 s, 72°C/60 s for 35 cycles. PCR produced a 750 bp amplicon from the mutated allele and a 520 bp amplicon from wild-type.

### Myofibre isolation

Mice aged 4–6 weeks were killed by cervical dislocation and the extensor digitorum longus (EDL) and/or soleus muscles removed from the hind limb. Muscles were incubated in 0.2% collagenase Type I/DMEM with 400 mM L-Glutamine (Sigma, Dorset, UK) and 1% (v/v) penicillin/streptomycin solution (Sigma, Dorset, UK) for 1.5 hour at 37°C. Collagenase was then inactivated and individual myofibres liberated by trituration, as described in detail elsewhere [18], [19]. Selected myofibres were free of capillaries or residual connective tissue. 15 or more isolated myofibres from at least 3 mice per genotype were analyzed for each experiment.

In order to determine the total number of myonuclei, myofibres were immunostained for Pax7 (to identify satellite cells, [20]) and 4,6-diamidino-2-phenylindole (DAPI) to visualize all nuclei (both myonuclei and satellite cells). EDL myofibres were isolated from 5 wild-type, 5 *lmna* ^−/+^ and 7 *lmna* ^−/−^ age-matched mice and multiple myofibres analyzed per type.

### Antibodies and immunostaining

Myofibres were fixed in 4% paraformaldehyde/PBS for 10 minutes, permeabilised with 0.5% (v/v) Triton X-100 in PBS and then blocked using 10% (v/v) goat serum and 10% (v/v) swine serum (DakoCytomation, Ely, UK) in PBS.

Primary antibodies used were monoclonal mouse anti-Pax7 (Developmental Studies Hybridoma Bank, Iowa, USA), polyclonal rabbit anti-acetylated (K9 and K14) -Histone H3 (Millipore, Watford, UK) and monoclonal rabbit anti-PABPN1 (clone EP3000Y, Epitomics, CA, USA). Primary antibodies were visualized with species-specific highly adsorbed Alexafluor-conjugated secondary antibodies (Cell Signalling, MA, USA) before mounting on slides with VECTASHIELD Mounting Medium containing 1.5 mg/ml of DAPI (Vector Laboratories, Peterborough, UK).

Images were acquired using a LSM 5 *EXCITER* confocal microscope equipped with a water immersion LD C-Apochromat 40x/1.1 W Corr objective with acquisition software ZEN 2007 LSM (Zeiss), or a Zeiss Axiophot 200 M microscope with a Charge-Coupled Device (Zeiss AxioCam HRm). Images were adjusted globally for brightness and contrast and assembled into figures using Adobe Photoshop CS.

### Transmission electron microscopy

Six EDL and soleus muscles from age-matched (35±1 days) wild-type (n = 3) and *lmna*-null (n = 3) mice were fixed in 2.5% phosphate buffered glutaraldehyde (pH 7.3, 0.1 M) for 4 hours at 4°C. The samples were then washed and post-fixed in 1% OsO_4_ in 0.1 M Milloning's phosphate buffer pH 7.3 for 1.5 hour at 4°C. Dehydration in ascending grades of ethanol and resin impregnation at room temperature occurred prior to embedding in epoxy resin TAAB Premix Medium Resin Kit (TAAB Laboratories Equipment Limited, Berks, UK). Ultra-thin sections (∼80 nm thick) were stained with a saturated solution of uranyl acetate and 0.17% lead citrate in 0.1 N sodium hydroxide. At least 3 sections per muscle per mouse were analyzed and viewed at 75 Kv in a Hitachi H7600 Transmission Electron Microscope.

### Image analysis

Image J software (http://rsb.info.nih.gov/ij) was used to measure the length, width, area and perimeter of myonuclei on confocal images from multiple EDL myofibres. Images of myonuclei from soleus muscles acquired by TEM were also analyzed with Image J software to measure length and width of nuclei, together with the amount of highly condensed nuclear chromatin. TEM images from 22 myonuclei from 3 wild-type, and 32 myonuclei from 3 *lmna*-null mice (6 muscles per genotype) were analyzed.

### Muscle mechanical force evaluation

Three EDL and three soleus muscles were dissected from wild-type, *lmna* ^−/+^ and *lmna* ^−/−^mice aged 4 and 5 weeks (3 mice per genotype per age) and their contractile properties were measured in an experimental chamber filled with an oxygenated Krebs-Henseleit solution at 25°C. The chamber was perfused continuously with 95% O_2_/5% CO_2_. One end of the muscle was attached to the force transducer (300B, Aurora Scientific Inc., Ontario, Canada) and the other end to a fixed steel hook. The muscles were stimulated by an electric field generated between two platinum electrodes placed longitudinally on either side of the muscle. Square wave pulses 0.2 ms in duration were generated by a stimulator to produce a maximum isometric twitch force. Muscles were adjusted to the optimum length for the development of maximum isometric tetanic contraction. After the experiment, the muscle was blotted dry and weighed, and the mean cross-sectional area was calculated assuming a muscle density of 1.06 mg mm^−3^ [21]. The muscle specific force at optimum length was expressed as the maximum isometric tetanic force per unit cross-sectional area of muscle.

All statistics are given as mean ± SEM. Unpaired *t*-test was used, where *p*<0.05 (two-tailed) was considered statistically significant.

## Results

### Myofibres from *lmna*-null mice have fewer myonuclei and satellite cells

We used the EDL and soleus muscles from the crural hind limb, since lower leg muscles are among those more severely affected in human EDMD patients [22]. In addition, the EDL is mainly composed of fast type IIx and IIb fibre types, while soleus comprises slow type I and fast type IIa fibres [23], [24], allowing assessment of the major extrafusal muscle fibre types in mouse.

To first determine if *lmna*-null mice had as many myonuclei and satellite cells as controls, myofibres were isolated from the EDL muscle of *lmna* ^−/−^, *lmna* ^−/+^ and *lmna* ^+/+^ (wild-type) mice and immunostained for Pax7 to identify satellite cells, then counterstained with DAPI to distinguish myonuclei [25]. EDL myofibres from *lmna* ^−/−^ mice contained significantly fewer myonuclei (201.1±3.8 versus 289.5±7.7 in wild-type) and satellite cells (3.3±0.2 versus 4.6±0.3 in wild-type) than either *lmna* ^−*/+*^ or wild-type; which had similar numbers (Table 1). Interestingly, the ratio of satellite cell number to total nuclei number per myofibre, remained constant at ∼1.6±0.1 for each genotype (Table 1).

**Table 1:** Total nuclei and satellite cells in EDL myofibres from *lmna*-null mice.

	Total nuclei per EDL myofibre	Satellite cells per EDL myofibre	Satellite cell/total nuclei ratio
**Wild-type** (n = 67)	289.5±7.7	4.6±0.3	1.6±0.1
***lmna*** **^−^** ^***/+***^ (n = 68)	268.9±5.4	4.4±0.6	1.6±0.1
***lmna^−/−^*** (n = 97)	201.1±3.8^*^	3.3±0.2^*^	1.6±0.1

20 myofibres from 3 mice per genotype were analyzed. Total number of myofibres analyzed is indicated in parenthesis. Values are mean ± SEM. An asterisk denotes *p*<0.01 compared to wild-type using Student's t-test.

### Myonuclear morphology is abnormal in *lmna*-null mice

**Figure 1:**
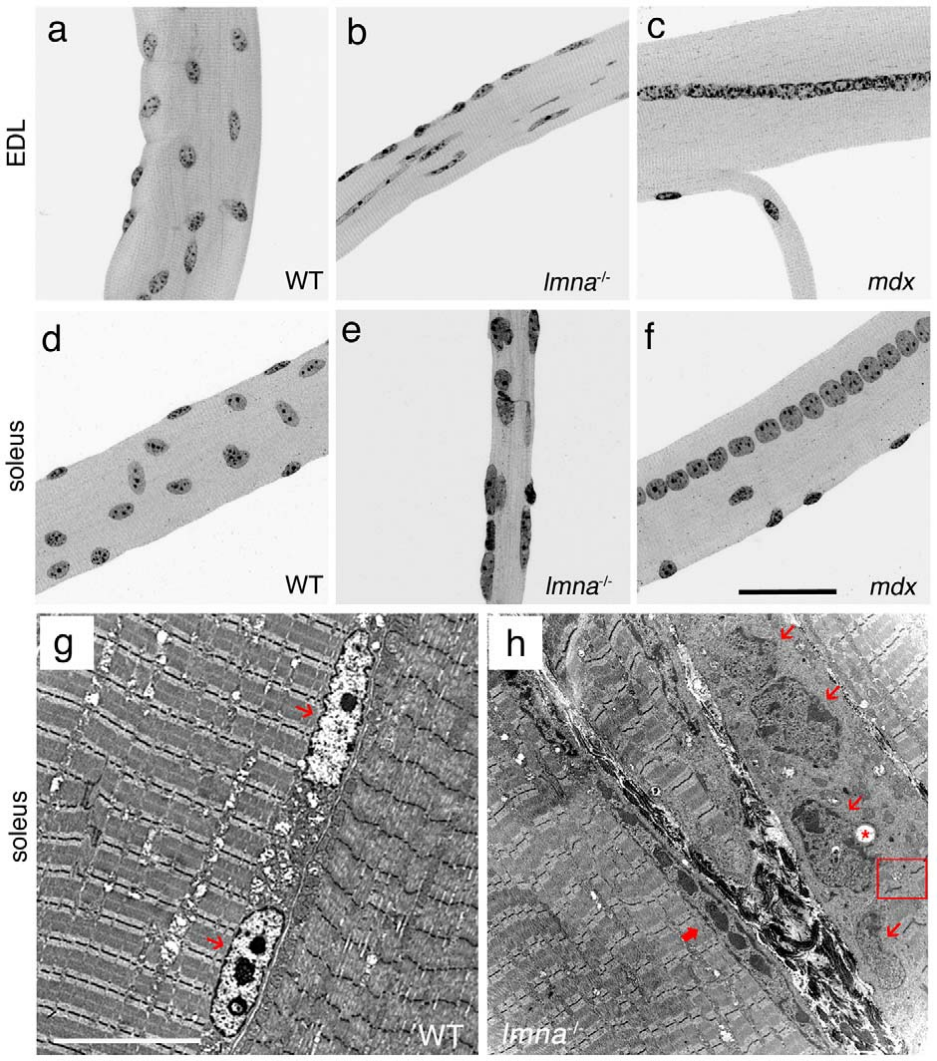
Myonuclear morphology is abnormal in *lmna*-null mice. DAPI staining of representative EDL and soleus myofibres from wild-type (WT) *lmna^+/+^*mice show that myonuclei are evenly distributed and have similar shape, size and heterochromatin content (**a and d**). By contrast, myonuclei in *lmna* ^−/−^ myofibres are unevenly distributed, with variable size and shape, and heterogeneous chromatin content and distribution (**b and e**). Myofibres isolated from the *mdx* mouse model of DMD contain myonuclei of a more regular size, shape and heterochromatin organization (**c and f**). Unlike in *lmna* ^−/−^ myofibres, myonuclei in *mdx* mice are often located in a chain in the centre of the myofibre, indicative of a recent regenerative event (**c and f**). Representative TEM images of longitudinal sections of soleus muscle from wild-type *lmna^+/+^* (**g**) and *lmna* ^−/−^ (**h**) mice. WT myonuclei (thin red arrows) are regularly shaped, and have an even layer of highly condensed chromatin around the nuclear rim, in addition to centrally located condensations (**g**). Myonuclei (thin red arrows) from *lmna* null mice are irregularly shaped and have disorganized chromatin throughout with occasional vacuoles (**h** - red *). A thick red arrow indicates an abnormally elongated myonucleus. Note connective tissue between myofibres and the disruption of the sarcomeric arrangements near the abnormal myonuclei (red open square). Scale bar for (**a–f**) is 50 µm and 10 µm for (**g and h**).

To next examine the morphology and chromatin distribution in myonuclei, myofibres were isolated from both the EDL and soleus muscles of *lmna* ^−/−^, *lmna* ^−/+^ and *lmna* ^+/+^ (wild-type) mice, together with those from *mdx* mice (a model of DMD [26], [27]). In both *lmna* ^−/+^ and wild-type mice, 98% of myonuclei were of a regular oval shape, similar in size and evenly distributed along the entire length of the myofibre (Figure 1a and 1d). The long and short axes of myonuclei in wild-type EDL myofibres were 13.1±0.2 µm and 6.8±0.1 µm respectively, and the length/width ratio was 2±0.1. Their contour ratio (4π area/perimeter^2^ - a measure of how close to round a structure is) was 0.8±0.01. By contrast, myonuclei in *lmna* ^−/−^ mice varied greatly in size, were irregularly shaped, and often were unusually elongated along the long axis of the myofibre (Figure 1b and 1e). The long and short axes of myonuclei of *lmna*-null EDL myofibres were 16.1±1.2 µm and 4.6±0.3 µm, respectively, and their length/width ratio was 4.5±0.7 (*p*<0.01 compared to wild-type) with a contour ratio of 0.6±0.02. All *lmna*-null myofibres analyzed had regions containing abnormal myonuclei and DAPI-stained fragments, but there was no evidence of myofibre branching or organized chains of centrally located myonuclei, indicative of muscle regeneration (Figure 1b and 1e). While myofibres from the soleus of *lmna*-null mice clearly contained many myonuclei with an abnormal morphology (Figure 1e), it was often difficult to delimit individual myonuclei to get a representative sample for detailed measurements, as performed for EDL myofibres. There was also a higher variability in EDL and soleus myofibre length and diameter between *lmna* ^−/−^ mice compared to those from either *lmna* ^−/+^ or wild-type (data not shown).

### Condensed chromatin amount and distribution are altered in myonuclei lacking lamin A/C

DAPI binds to double-stranded DNA and is routinely used to examine condensed chromatin (heterochromatin) distribution [28]. In wild-type EDL and soleus myofibres, the DAPI staining was similar in all myonuclei, with strongly stained chromatin regions regularly distributed throughout the myonucleus (Figure 1a and 1d). By contrast, *lmna*-null mice exhibit differential DAPI staining between myonuclei, with variable amounts of irregularly distributed, and highly condensed, chromatin (Figure 1b and 1e).

To increase resolution, we used Transmission Electron Microscopy (TEM) on ultra-thin sections of soleus muscle from wild-type *lmna^+/+^* and *lmna*-null mice (Figure 1g and 1h). The ratio of nuclear length to width showed that myonuclei from soleus were significantly more elongated (7.1±0.8 compared to 2.7±0.2 - Table 2). Myonuclei from wild-type mice possessed a condensed chromatin layer directly inside the nuclear membrane. In addition, there were one or more round central clumps of condensed chromatin, indicative of nucleoli, together with occasional smaller accumulations (Figure 1g). In ∼85% of *lmna*-null myonuclei analyzed by TEM, such highly organized chromatin distribution was lost (Figure 1h). Furthermore, it was no longer completely juxtaposed to the inner nuclear membrane. Such a distribution of heterochromatin can be a sign of DNA fragmentation and a key feature of the early stages of apoptosis [29]. Measuring the area of the myonucleus occupied by electron dense chromatin, revealed that it was significantly higher in *lmna* ^−/−^ mice (63.4±2.2% versus 49.4±2.1% in wild-type - Table 2). Moreover, ∼24% of the analyzed myonuclei contained vacuoles (labelled with an asterisk in Figure 1h). Interestingly, myofibres often displayed disorganized sarcomeres in close proximity to the most affected myonuclei (Figure 1h - residual sarcomeres in an highly disorganized area are shown in the red box).

**Table 2:** Morphological and chromatin content alterations in soleus myonuclei of *lmna*-null mice.

	Length (µm)	Width (µm)	Length/Width ratio	Percentage of nuclear volume occupied by condensed chromatin
**Wild-type myonuclei** (n = 22)	8.4±0.5	3.4±0.3	2.7±0.2	49.4±2.1%
***lmna*** **-null myonuclei** (n = 32)	12.5±1.1	2.2±0.2	7.1±0.8^*^	63.4±2.2%^*^

7 myonuclei from 3 mice per genotype were analyzed by TEM. Total number of myonuclei analyzed is indicated in parenthesis. Values are mean ± SEM. An asterisk denotes *p*<0.01 compared to WT using Student's *t*-test.

### Abnormal myonuclei are not a hallmark of all dystrophic muscle

To determine whether our observations on the myonuclei of *lmna*-null myofibres were a general feature of dystrophic muscle, or specific to the dystrophic phenotype associated with a lack of lamin A/C, we also isolated myofibres from the *mdx* mouse model of DMD [26], [27]. Myofibres from EDL and soleus of *mdx* mice had highly variable lengths and diameters (data not shown), more so than *lmna* ^−/−^ myofibres, and were occasionally split and branched (Figure 1c and 1f). However, myonuclei had a regular shape and size, and were evenly distributed, although there were many areas containing chains of centrally located myonuclei (Figure 1c and 1f), indicative of muscle regeneration. Moreover, DAPI staining of *mdx* myonuclei revealed an overtly normal chromatin distribution.

#### Abnormal myonuclei accumulate at the myotendinous junction

**Figure 2:**
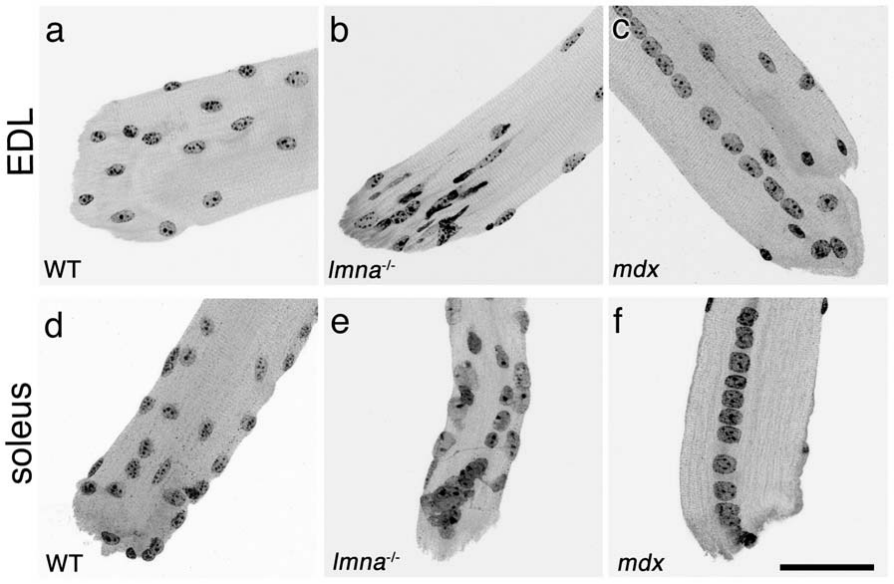
Myonuclei cluster at the myotendinous junctions of *lmna^−/−^* myofibres. DAPI staining at the myotendinous junction from wild-type (WT) *lmna^+/+^* EDL and soleus myofibres show that myonuclei are evenly distributed, with similar shape, size and chromatin organization (**a and d**). Myonuclei tend to cluster at the myotendinous junction from *lmna* ^−/−^ mice, and are unevenly distributed with varying sizes, shapes and condensed chromatin content (**b and e**). Myonuclei of the myotendinous junction from *mdx* mice have an overtly normal morphology, although often in centrally located chains that continue to the end of the myofibre (**c and f**). Scale bar 50 µm.

The myotendinous junction is a specialised structure where the muscle connects to the tendon, and is the principal site of longitudinal force transmission across the muscle cell membrane [30], [31]. Myonuclei at the myotendinous junction of wild-type *lmna^+/+^* myofibres were indistinguishable from those located elsewhere along the fibre (Figure 2a and d). By contrast, many myonuclei at the myotendinous junction of *lmna* ^−/−^ mice were clearly structurally abnormal, with aberrant chromatin accumulations (Figure 2b and e). Measuring the 10 myonuclei closest to the myotendinous junction (from at least 5 myotendinous junctions from 5 mice per genotype) we found that the average length and width of wild-type myonuclei were 11.1±0.2 µm and 6.1±0.1 µm, respectively; whereas the average length and width of the 10 *lmna*-null myonuclei closest to the myotendinous junction were 16.5±0.9 µm and 4.7±0.2 µm. Thus, the length/width ratio for *lmna*-null myotendinous junction myonuclei was 4.0±0.3, significantly different (*p*<0.01) from the wild-type ratio of 1.9±0.1. The average contour ratio was 0.6±0.02 for *lmna*-null myonuclei at the myotendinous junction versus 0.8±0.01 for wild-type (*p*<0.01). Importantly, ∼85% of myofibres from *lmna*-null mice had myotendinous junctions at which myonuclei were clustered (Figure 2b and e). Again, we examined whether this was a common feature of muscular dystrophy. Myotendinous junctions of *mdx* myofibres did not accumulate myonuclei, and when present, chains of centrally located myonuclei often remained in register until the extremity of the myotendinous junction (Figure 2c and f).

### Heterogeneous transcriptional activity of myonuclei in *lmna*-null mice

Abnormal chromatin distribution in the *lmna-*null mice may indicate compromised transcription. Histones can be epigenetically modified by site-specific combinations of phosphorylation, acetylation and methylation, which correlate with specific biological readouts, such as transcriptional activation or repression, chromatin remodelling or stabilization [32]. In particular, acetylation of histones H3 and H4 positively correlates with active gene transcription [33], [34].

**Figure 3:**
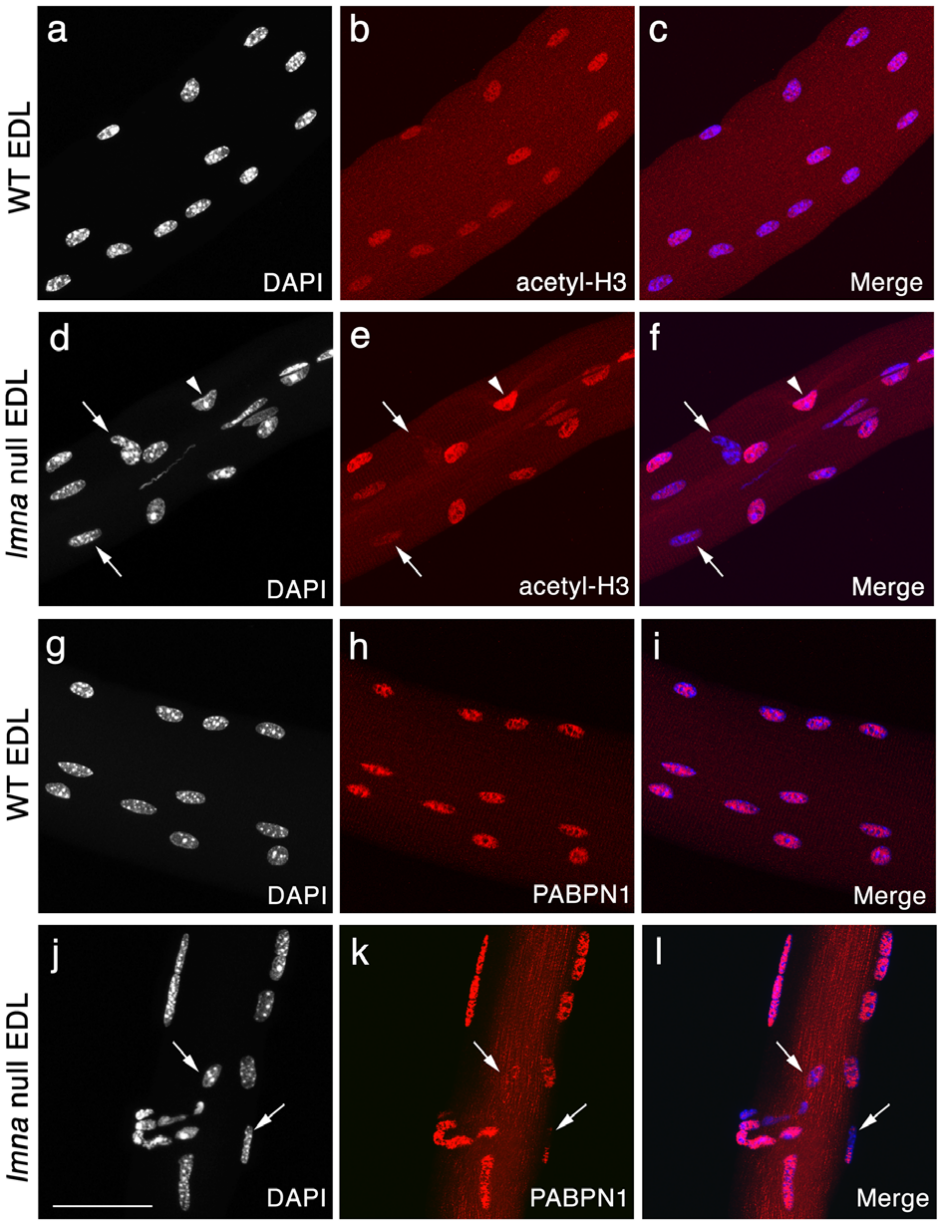
Variable transcriptional activity and mRNA processing between myonuclei of *lmna*-null mice. Representative images of wild-type (WT) *lmna^+/+^* EDL myofibres, immunostained for acetyl-histone H3 (red) and counter-stained with DAPI (white), with merged images (**a–c**). Myonuclei in WT myofibres have near uniform acetyl-histone H3 immunostaining, suggesting similar transcriptional activity. By contrast, immunostaining of *lmna* ^−/−^ EDL myofibres showed that myonuclei clearly have varying acetyl-histone H3 levels, indicating heterogeneous transcriptional activity, with some immunostaining at near background levels (**d–f -** arrows) while others appear hyperacetylated (**d–f -** arrowhead). WT myonuclei also show virtually homogeneous PABPN1 immunostaining (**g–i**)**,** while in many myonuclei of *lmna* ^−/−^ EDL, PABPN1 is either reduced, or absent (**j–l -** arrows). Scale bar 50 µm.

As an assessment of transcriptional activity of myonuclei, we immunostained for acetylated histone H3 (K9 and K14). Largely homogeneous immunostaining of acetyl-H3 was observed in myonuclei from freshly isolated EDL myofibres of wild-type *lmna^+/+^* mice, indicating coordinated transcriptional activity (Figure 3a-c). By contrast, myonuclei in *lmna*-null myofibres had heterogeneous levels of histone H3 acetylation, with some myonuclei virtually unstained, while others exhibited normal, or even apparently increased, levels of immunostaining. These varying levels of histone H3 acetylation indicate that transcriptional activity differs between individual, often adjacent, myonuclei (Figure 3d–f). Approximately 50% of myonuclei were clearly misshapen in EDL *lmna*-null myofibres, compared to ∼2% for those of wild-type. While severely misshapen myonuclei were most likely to exhibit background levels of acetyl-histone H3 immunostaining (∼26% of the 50% misshapen, so ∼13% of total nuclei were hypoacetylated), there were also a significant number of overtly morphologically normal myonuclei that were hypoacetylated (∼7%, i.e. ∼3.5% of total). Western Blot analysis of EDL myofibres showed that global acetyl-histone H3 levels were significantly increased (∼2.5 fold) in *lmna*-null myofibres compared to wild-type (data not shown).

Next, we assessed the state of the transcriptional machinery: poly(A) binding protein nuclear 1 (PABPN1) is an abundant nuclear protein that is part of the polyadenylation complex and integral for completion of messenger RNA maturation [35]. Myonuclear PABPN1 immunostaining was virtually homogeneous in wild-type *lmna^+/+^* myofibres (Figure 3g–i), whereas it varied greatly between individual, often adjacent, myonuclei in *lmna*-null myofibres (Figure 3j–l). Together with the epigenetic changes indicated by variable histone acetylation, the distribution of PABPN1 further indicates that gene expression is deregulated in the absence of lamin A/C. The wide variation in transcriptional activity both between individual myonuclei and myofibres is consistent with the transcriptional deregulation observed in patient muscle [10]. Unfortunately, we were unable to co-immunostain for acetyl-histone H3 and PABPN1 because both antibodies were raised in rabbit, and were of the same isotype.

**Figure 4:**
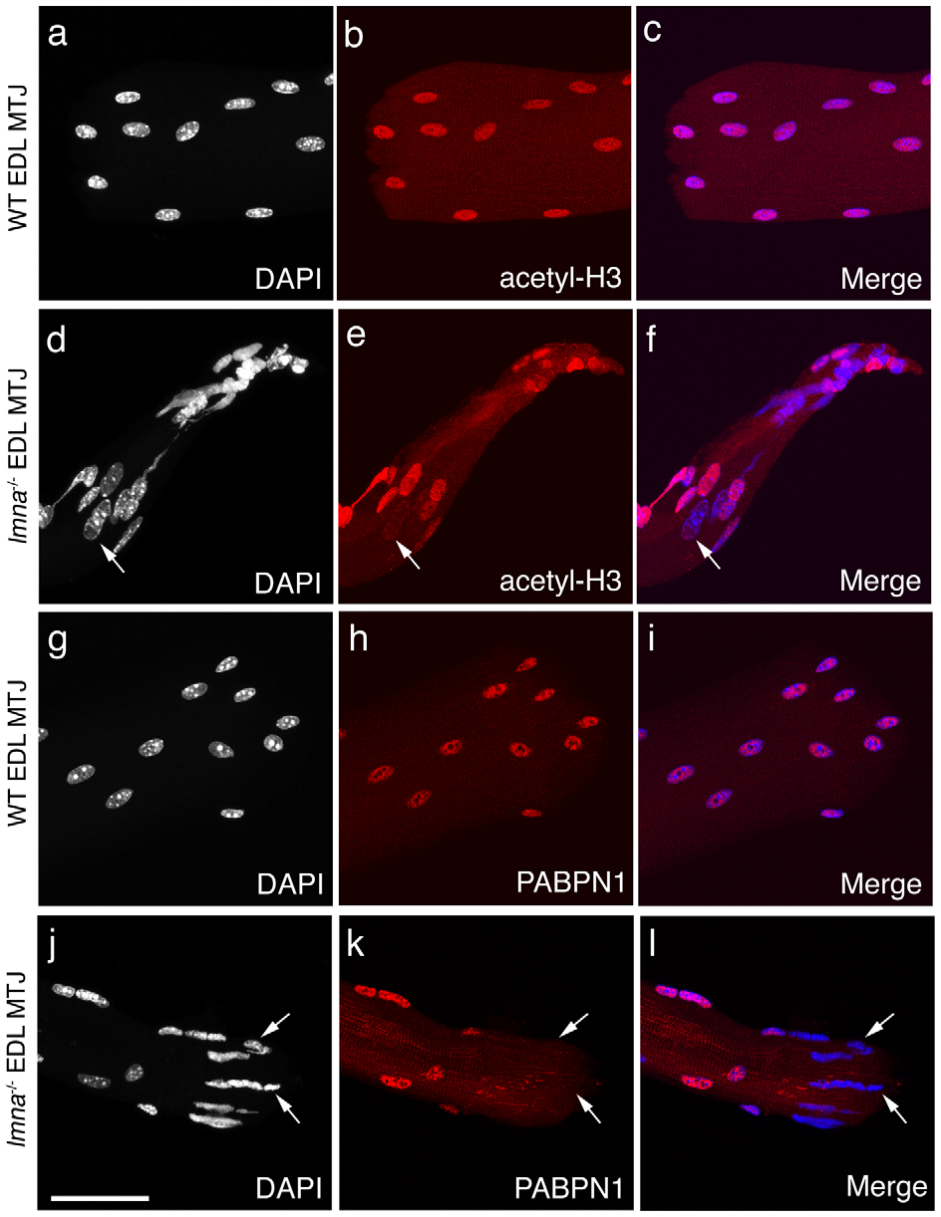
Transcriptional activity and mRNA processing are often impaired in myonuclei at the myotendinous junctions of *lmna*-null mice. Representative images of wild-type (WT) *lmna^+/+^* EDL myofibres immunostained for acetyl-histone H3 (red) and counter-stained with DAPI (white), with merged images (**a–c**). Myonuclei at the myotendinous junction (MTJ) in WT myofibres have near homogeneous acetyl-histone H3 immunostaining, as observed along the myofibre, indicating similar levels of transcriptional activity. By contrast, immunostaining of *lmna* ^−/−^ EDL myofibres revealed that myonuclei at the myotendinous junctions clearly had varying acetyl-histone H3 levels, indicating heterogeneous transcriptional activity, ranging from virtually inactive (background levels – arrow in **d–f**), to hyperacetylated, myonuclei (**d–f**). WT myonuclei also exhibit regular and homogeneous PABPN1 immunostaining at the myotendinous junction (**g–i**). In myonuclei at the myotendinous junctions of *lmna* ^−/−^ EDL however, varying PABPN1 levels are observed, with many being virtually unstained (**j–l** - arrows), indicating an impairment of mRNA processing and maturation. Scale bar 50 µm.

Consistent with myonuclei distributed along the myofibre, myonuclei at the myotendinous junctions in wild-type *lmna^+/+^* mice had near homogeneous immunostaining for acetyl-H3 (Figure 4a–c) and PABPN1 (Figure 4g–i). However, myonuclei at the myotendinous junctions of *lmna*-null mice exhibited heterogeneous immunostaining, with a majority being hypoacetylated (Figure 4d–f) or having a marked reduction in PABPN1 protein levels (Figure 4j–l).

### The structure of the myotendinous junction is severely perturbed in *lmna*-null mice

**Figure 5:**
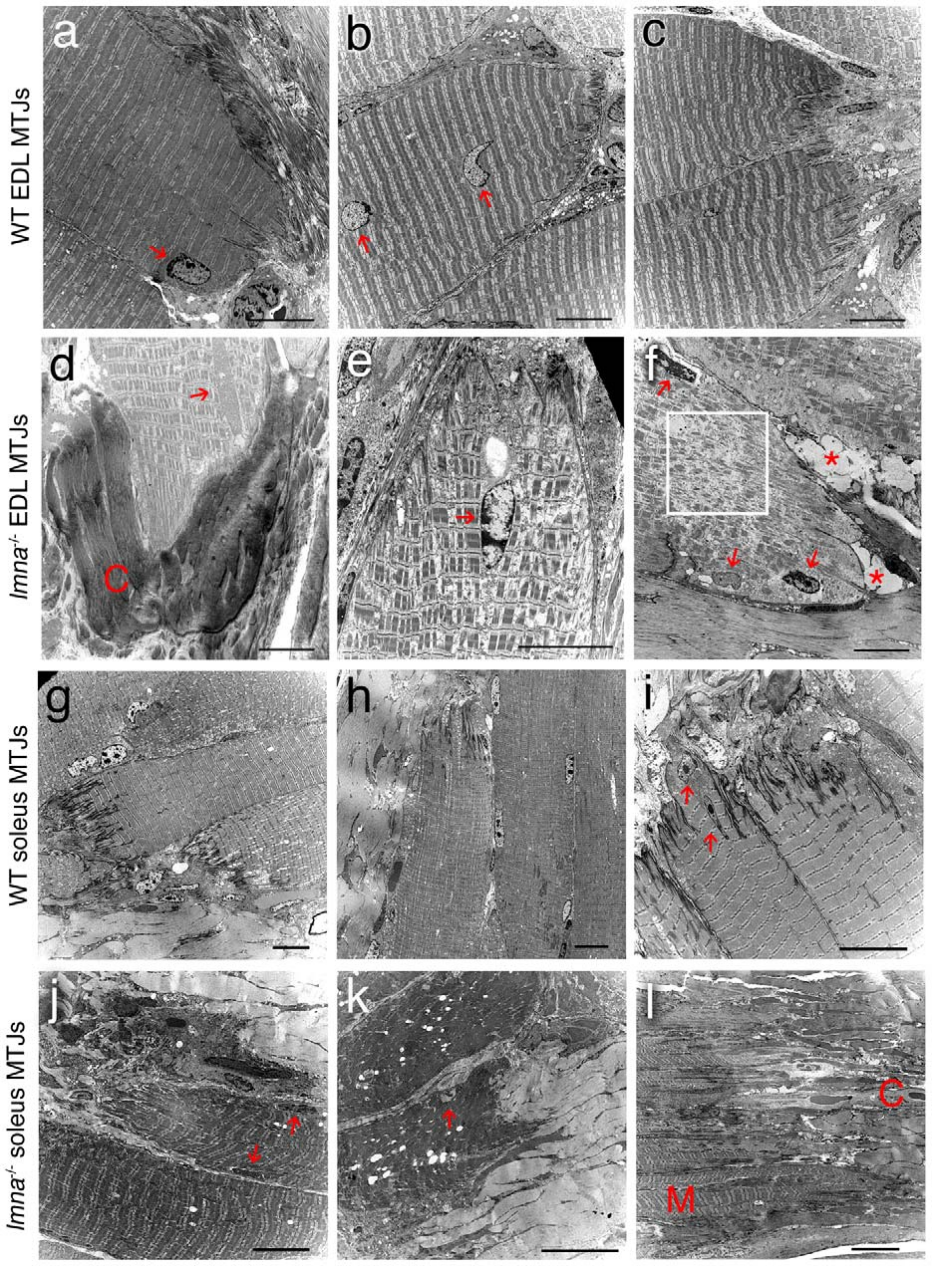
Myotendinous junction structure is abnormal in *lmna^−/−^* mice. Representative TEM images of longitudinal sections of myotendinous junction (MTJ) from EDL (**a–c**) and soleus (**g–i**) myofibres of wild-type (WT) *lmna^+/+^* mice. Myonuclei (red arrows) and sarcomere organisation appear normal. Note the extensive inter-digitations between the myofibre and tendon. TEM images of longitudinal sections of myotendinous junctions from EDL (**d–f**) and soleus (**j–l**) myofibres from *lmna* ^−/−^ mice. Myonuclei with abnormal shape, size and chromatin organization are evident (red arrows). There is a lack of inter-digitations, abnormal connective tissue (C in panel **d**) and fat accumulations (asterix in panel **f**) and sarcomeric disorganisation (boxed area in panel **f**). Myotendinous junctions in soleus muscle are particularly badly affected (**j–l**). The tissue architecture in (**l**) is so disrupted that the muscle region (M) and the connective tissue region (C) are barely distinguishable. Scale bar in each image equals 10 µm.

Examining the ultrastructure of both EDL and soleus muscles using TEM, we found that the myotendinous junctions from wild-type *lmna^+/+^* mice had myonuclei with normal morphology, a regular sarcomeric organization, and marked interdigitations between the myofibre and tendon (Figure 5a–c and 5g–i). In *lmna* ^−/−^ mice however, ∼90% of myotendinous junctions in EDL (Figure 5d–f) and all examined in soleus (Figure 5j–l) exhibited structural abnormalities including: myofibril loss in the proximity of the plasmalemma (∼92% EDL and ∼95% soleus - Table 3); increased fibrotic and adipose deposition (∼83% EDL and ∼92% soleus - Table 3); reduced and irregular interdigitation (∼75% EDL and ∼82% soleus - Table 3) and presence of vacuoles (∼42% EDL and ∼63% soleus - Table 3). Disorganised sarcomeres were regularly observed at the myotendinous junction, which was more pronounced in the proximity of abnormal myonuclei. Myotendinous junctions in soleus were more disrupted than those of the EDL (Table 3), often showing a complete loss of the organized transition between muscle and tendon (as illustrated in Figure 5l).

**Table 3:** Quantification of myotendinous junction abnormalities in *lmna*-null mice.

	Myofibril loss	Increased fibrosis	Decreased interdigitation	Vacuoles
**Wild-type EDL myotendinous junction**	2/33 (6.1%)	1/33 (3.0%)	2/33 (6.1%)	2/33 (6.1%)
***lmna-*** **null EDL myotendinous junction**	22/24 (91.7%)	20/24 (83.3%)	18/24 (75.0%)	10/24 (41.7%)
**Wild-type soleus myotendinous junction**	2/35 (5.7%)	0/35 (0%)	1/35 (2.9%)	2/35 (5.7%)
***lmna*** **-null soleus myotendinous junction**	38/38 (100%)	35/38 (92.1%)	31/38 (81.6%)	24/38 (63.1)

TEM images of 33 wild-type EDL myotendinous junctions, 24 *lmna*-null EDL myotendinous junctions, 35 wild-type soleus myotendinous junctions and 38 *lmna*-null soleus myotendinous junctions were acquired from a minimum of 7 myotendinous junctions from 3 mice per genotype.

### *Lmna-*null mice have delayed growth and decline in weight after a month of age

*Lmna*-null mice show post-natal growth retardation with weight ∼50% of their wild-type littermates at 28 days of age [14]. Similarly, we found that wild-type and *lmna* ^−/+^ mice were indistinguishable 24 days after birth, whereas *lmna* ^−/−^ mice were approximately half their size (6.3±1.2 g). Both wild-type and *lmna* ^−/+^ mice increased their body mass by ≥0.5 g per day, whereas *lmna*-null body mass increased by only ∼0.2 g per day, and reached a maximum by day 29 to 32, after which it declined by ∼0.3 g per day. By day 37 *lmna* ^−/−^ mice were less than one third the size of wild-type and heterozygous littermates (6.3±0.9 versus 20.6±0.7 and 20.2±0.9 g respectively). Generally, between day 34 and 37 *lmna*-null mice stopped moving and were sacrificed.

### *Lmna-*null muscles generate less force

To examine if the structural and transcriptional changes we identified correlate with compromised muscle function, we measured force generation in EDL and soleus muscles at 4 and 5 weeks of age. Muscle contractile properties were tested in terms of muscle specific force, which was measured at 25°C and calculated by dividing maximum isometric tetanic force by mean muscle cross-sectional area. Three EDL and three soleus muscles removed from wild-type *lmna^+/+^*, *lmna* ^−/+^ and *lmna* ^−/−^ mice (n = 3 per age group) aged 4 weeks (mice still growing and active) and compared to those from 5 weeks old mice (mice no longer growing, often barely active). EDL and soleus muscle mass was reduced in *lmna*-null mice, in accordance with the reduced total body mass. Moreover, the muscle mass actually declined between 4 and 5 weeks, with EDL and soleus of *lmna*-nulls ∼80% of wild-type and heterozygous at 4 weeks, and ∼35% by 5 weeks.

**Figure 6:**
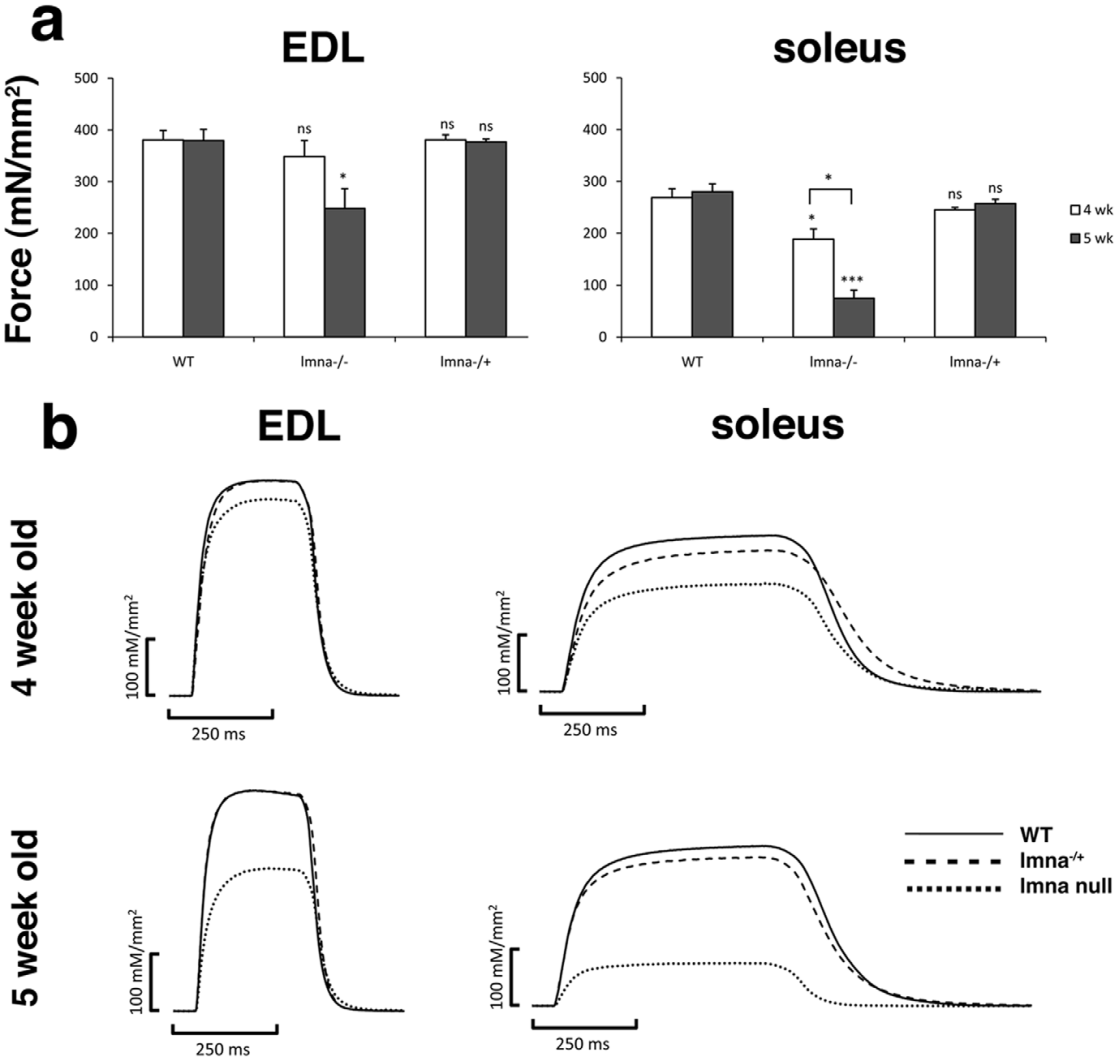
Force generation is impaired in EDL and soleus muscles lacking lamin A/C. Muscle specific force produced by EDL (left panel) and soleus (right panel) from wild-type (WT) *lmna^+/+^*, *lmna* ^−/−^ and *lmna* ^−/+^ mice at 4 (white bars) and 5 (grey bars) weeks of age (**a**). Generation of isometric tetanic force at optimim length by EDL and soleus from WT (—), *lmna* ^−/+^ (---) and *lmna* ^−/−^ (····) mice at 4 and 5 weeks of age (**b**). 3 muscles from 3 mice per genotype per age were analyzed. Values are mean ± SEM from three muscles. An asterisk denotes significance level using Student's *t*-test. *** *p*<0.001; * *p*<0.05; ^ns^ *p*>0.05. Each value is compared with that of age-matched WT. The muscle specific force was also significantly different for soleus muscles from *lmna* ^−/−^ mice between 4 and 5 weeks of age.

Soleus muscles from 4 week old *lmna* ^−/−^ mice showed a significant reduction in force generation, with muscle specific force only ∼70% of controls (Figure 6a). The muscle specific force in both EDL and soleus from 5 week old *lmna* ^−/−^ mice was significantly reduced. Mean *P*_0_ of soleus muscles was only ∼27% of age-matched controls, while it was ∼65% for the EDL (Figure 6a). There was no reduction in the force generation ability of muscles from *lmna* ^−/+^ mice at either age.

Importantly, the reduction in muscle specific force of *lmna*-null muscle was not accompanied by changes in force generation profile, with the soleus muscle still showing the force generation profile typical of a slow muscle and the EDL, that of a fast muscle (Figure 6b).

## Discussion

EDMD and LGMD1B are characterised by muscle weakness and wasting, with abnormal myonuclear morphology. However it is unclear how many myonuclei are actually affected, with estimates ranging from 10%–90% for those having morphological and/or chromatin irregularities [36]–[38]. These observations have been largely made using muscle sections however, which makes it is extremely difficult, if not completely impractical, to examine all myonuclei in a given myofibre: the basic functional unit of skeletal muscle. Furthermore, three dimensional myonuclear morphology and distribution are also not easily analysed. These limitations however, can be overcome by isolating entire myofibres where possible.

Myofibres from the *lmna*-null mouse model of A-EDMD varied in size, but were generally smaller than controls, containing ∼30% fewer myonuclei. Importantly, each myofibre contained myonuclei with abnormal morphology and chromatin distribution, which equated to ∼50% of the entire population in the EDL. These pathological hallmarks were even more widespread in the soleus, where altered chromatin distribution and increased compacted and clumped chromatin were evident using TEM. Interestingly, chains of centrally located myonuclei were not seen in adult *lmna-*null mice, while this hallmark of muscle regeneration is apparent in many myofibres of *mdx* mice [26].

Our findings are consistent with reports of the morphology and chromatin distribution in myonuclei from other skeletal muscles, cardiomyocytes and embryonic fibroblasts in *lmna*-null mice [15], [39] and in patients, where an overall decrease of condensed heterochromatin, focal loss of chromatin and increased clumping away from the myonuclear rim have been described [36]–[38], [40]. Similarly, ∼10% of myonuclei in the H222P mouse model of EDMD (containing a pathogenic point mutation at residue 222 in the *lmna* gene that causes familial A-EDMD and dilated cardiomyopathy in man) exhibit structural abnormalities and heterochromatin redistribution when examined using muscle sections [41]. Since similar changes occur in cardiomyocytes of *syne1* null mouse (lacking nesprin 1) [42], this indicates that disruption of different components of the LINC complex cause a common phenotype.

It is well established that lamin A/C have a crucial role in chromatin organization [43], [44] and gene transcription [45]. To understand how such morphological changes affect myonuclear function, we examined the transcriptional state of myonuclei by assessing both epigenetic modifications indicative of an active transcriptional state (histone H3 acetylation - [33], [34]) and PABPN1, a component of the transcriptional machinery. PABPN1 is an abundant nuclear protein that binds the poly(A) tail of pre-mRNA and is part of the polyadenylation complex through interactions with poly(A) polymerase and cleavage and adenylation specificity factor. PABPN1 stimulates the polymerization of the tail by poly(A) polymerase, thus controlling the length of the poly(A) tail [35].

Absence of lamin A/C caused heterogeneous levels of both acetylated histone H3 and PABPN1 between myonuclei, indicating that transcriptional activity varied and revealing a lack of coordinated transcriptional control. Importantly, altered acetyl-histone H3 patterns were not always linked to aberrant morphology, with many apparently ‘normal’ myonuclei being completely hypoacetylated, and so presumably transcriptionally inactive. Assessing global acetyl-histone H3 levels by Western blot, we found a significant increase (∼2.5 fold) in *lmna*-null myofibres compared to wild-type. Histone H3 hyperacetylated fibres have also been observed in the tibialis anterior muscles *of lmna*-null mice [46]. Therefore, the marked reduction/absence of acetyl-histone H3 from a significant number of *lmna*-null myonuclei was more than counterbalanced by the relative hyperacetylation in other myonuclei. Global epigenetic defects have been reported in myoblasts from A-EDMD patients carrying either the R377H or R545C mutations [47], [48], and also occur following over-expression of the A-EDMD-causing lamin A mutation R453W in C2 myoblasts [49]. Epigenetic modifications are also found in fibroblasts from patients with the premature aging disorder Hutchinson-Gilford progeria syndrome [50], indicating that it may be a feature common to laminopathies in general.

Lamin A/C not only binds chromatin and chromatin-associated proteins [51], [52], but can also associate with specific transcription factors. For example, lamin A/C-mediated c-Fos sequestration at the nuclear envelope and their interactions with ERK1/2, regulate AP1 (Activating Protein 1) activity [9], [53]. Thus regulation of transcription mediated by A-type lamins can operate through at least two inter-dependent mechanisms: firstly, through direct binding of chromatin and chromatin-associated proteins, the nuclear lamina can regulate chromatin positioning, promote silencing at the periphery and induce global epigenetic changes; secondly, by interaction with specific transcription factors and signal transduction components, it can mediate the fine tuning of tissue specific transcriptional programs and signalling pathways. Indeed, general disturbances of the transcriptome in A-EDMD and X-EDMD patient muscle have been reported using microarrays [10], with a number of signal transduction pathways being affected including Rb1/MyoD, MAPK/ERK, PI3K/Akt and TGFβ/Smad [10], [54]–[57].

A striking finding was that myonuclei of *lmna*-null mice not only clustered at the myotendinous junction, as previously reported [58], but were consistently abnormal, with irregular shape, chromatin distribution and importantly, reduced transcriptional activity. Thus, this myonuclear clustering may be a response to the compromised transcriptional activity of many myonuclei, and the necessity to reduce the average myonuclear domain. There were also more widespread structural defects at the myotendinous junction, including increased fibrotic and adipose tissue, loss of myofibrils and sarcomeric organization, the presence of vacuoles in the cytoplasm and a lack of interdigitation between the muscle fibre and tendon. EDMD is unique amongst muscular dystrophies in that contractures develop early, notably in the Achilles tendons and elbow, prior to any clinically significant muscle weakness. Since myotendinous junctions are the main point of force transmission between fibre and tendon, and the primary site of lesion in many muscle tears [30], [59], [60], we speculate that defects at the myotendinous junction could be in part responsible for the functional failure contributing to joint contractures. In particular, the lack of interdigitations and cytoplasmic splitting could significantly reduce the area over which the transmitted force is distributed [59], [60], and accumulation of connective tissue on the tendon side could cause stiffness and, ultimately, contractures. Myofibres from *mdx* mice have blunt myotendinous junctions, lacking the digit-like processes typical of wild-type myofibres [61], but we did not observe myonuclear clustering at the myotendinous junction in *mdx*. Thus, while these two mouse models share some pathophysiological changes at the myotendinous junction, myonuclei clustering is a specific feature of lamin A/C-deficient muscles. Interestingly, myonuclear positioning at the neuromuscular junction is also affected in *lmna*-null mice, and myonuclei also showed hyperacetylation of histone H3 lysine 9, a hallmark of muscle denervation [46].

The myotendinous junction is the essential force-transmitting component of the musculoskeletal system, and so their perturbed structure could affect force generation in muscles. While muscle specific force was reduced in both the EDL and soleus by 5 weeks of age, the soleus was more precociously and severely affected than EDL. This may correlate with the more widespread and greater disturbances in myonuclei and structure of the myotendinous junction in *lmna*-null soleus muscle. A drop in twitch and tetanic force generation in soleus and diaphragm muscle from *lmna*-null was previously reported [62]. However, soleus and diaphragm both consist of slow and fast muscle fibre types, and so we compared soleus with the EDL, which is almost exclusively composed of fast muscle fibre types [23]. It is important to note that the reduction in force generation was not accompanied by a change in the overall fibre type distribution, with the soleus still generating a typical tetanic force curve of a slow muscle and the EDL, that of a fast muscle. Although other factors probably contribute, such as the variable transcriptional status, the perturbed myotendinous junction structure is likely to be a significant factor.

In conclusion, analyzing myofibres from the *lmna*-null mouse model of A-EDMD revealed that compromised myonuclear structure and transcriptional deregulation were widespread. Abnormal myonuclei accumulate at the myotendinous junction, the structure of which is clearly perturbed. Combined, these changes presumably result in the rapid decline in force generation. Therefore, myonuclear and myotendinous junction dysfunction may act synergistically to produce the dystrophic phenotype in A-EDMD.
